# Evaluation of the impact of a brief educational message on clinicians’ awareness of risks of ionising-radiation exposure in imaging investigations: a pilot pre-post intervention study

**DOI:** 10.1186/s12913-019-4712-y

**Published:** 2019-11-14

**Authors:** Ben Young, Jo Cranwell, Andrew W. Fogarty, Rob Skelly, Nigel Sturrock, Mark Norwood, Dominick Shaw, Sarah Lewis, Tessa Langley, Peter Thurley

**Affiliations:** 1 Division of Epidemiology and Public Health, Clinical Sciences Building, Nottingham City Hospital, University of Nottingham, Hucknall Road, Nottingham, NG5 1PB UK; 2 Department for Health, University of Bath, Claverton Down, Bath, BA2 7AY UK; 3 Royal Derby Hospital, Uttoxeter Road, Derby, DE22 3NE UK; 4 NIHR Nottingham Biomedical Research Centre, Clinical Sciences Building, Nottingham City Hospital, University of Nottingham, Hucknall Road, Nottingham, NG5 1PB UK

**Keywords:** Computed tomography, Radiation risks, Radiation protection, Clinician knowledge, Clinician education

## Abstract

**Background:**

In the context of increasing availability of computed tomography (CT) scans, judicious use of ionising radiation is a priority to minimise the risk of future health problems. Hence, education of clinicians on the risks and benefits of CT scans in the management of patients is important.

**Methods:**

An educational message about the associated lifetime cancer risk of a CT scan was added to all CT scan reports at a busy acute teaching hospital in the UK. An online multiple choice survey was completed by doctors before and after the intervention, assessing education and knowledge of the risks involved with exposure to ionising radiation.

**Results:**

Of 546 doctors contacted at baseline, 170 (31%) responded. Over a third (35%) of respondents had received no formal education on the risks of exposure to ionising radiation. Over a quarter (27%) underestimated (selected 1 in 30,000 or negligible lifetime cancer risk) the risk associated with a chest, abdomen and pelvis CT scan for a 20 year old female. Following exposure to the intervention for 1 year there was a statistically significant improvement in plausible estimates of risk from 68.3 to 82.2% of respondents (*p* < 0.001). There was no change in the proportion of doctors correctly identifying imaging modalities that do or do not involve ionising radiation.

**Conclusions:**

Training on the longterm risks associated with diagnostic radiation exposure is inadequate among hospital doctors. Exposure to a simple non-directional educational message for 1 year improved doctors’ awareness of risks associated with CT scans. This demonstrates the potential of the approach to improve knowledge that could improve clinical practice. This approach is easily deliverable and may have applications in other areas of clinical medicine. The wider and longer term impact on radiation awareness is unknown, however, and there may be a need for regular mandatory training in the risks of radiation exposure.

## Background

The number of annual computerised tomography (CT) scans performed on NHS patients in England increased from 3.3 million in 2012–13 to 5.4 million in 2018–19 [1]. Demand for CT scans can vary by geographic region and between referring doctors [2, 3], suggesting a proportion of scans may be avoidable. The main serious longterm health consequence of exposure to ionising radiation in a CT scan is the lifetime risk of cancer [4], with plausible estimates lying between one in 300 and one in 3000. Female and younger aged patients have greater susceptibility to harm [5]. There is an approximately threefold increase in the rate of use of CT scans over the period of transition from paediatric to adult care [6], indicating that paediatric doctors may use effective strategies to reduce exposure to ionising radiation. Although UK regulations and professional guidelines dictate that patients must be protected from unnecessary exposure to radiation [7–9], this assumes that requesting clinicians know that these imaging modalities involve radiation exposure and of the consequences of this; however previous studies have highlighted deficits in awareness [10–15]. As access to CT scans becomes more available in the UK and elsewhere, the threshold for requesting them may decrease, hence improving awareness among doctors of the risk of exposure to CT scans on future health is important as alternative safer options might be overlooked.

One strategy to improve efficiency and reduce harm in healthcare involves improving clinicians’ knowledge of the cost of their decisions, using non-judgemental ‘nudges’ based on behavioural insight theory [16]. This approach aims to improve decision-making without restricting freedom of choice. Brief educational messages can be presented passively without the alert fatigue associated with traditional interruptive warnings and reminders. Financial cost information delivered in this way has been shown to significantly reduce demand for blood tests by hospital clinicians [17], and avoidable ionising radiation exposure can be considered another ‘cost’ associated with the use of diagnostic testing. In this study we implemented a simple non-directional educational intervention about the associated risk of CT scans that was associated with a significant reduction of 4.6% in use of CT scans during the 12 month intervention period compared to a control group [18].

It is important to assess the baseline awareness of clinicians’ knowledge of the health impacts of ionising radiation exposure, as well as if the educational intervention actually improved doctors’ awareness of the risk of CT scans and alternative imaging modalities. We aimed to measure change in doctors’ knowledge of associated risks of exposure to CT scans and other imaging procedures before the intervention and after exposure to it for 1 year.

## Methods

We used a repeated cross-sectional study design to evaluate the intervention. The setting was a busy teaching hospital in the UK. Data were collected as part of a larger study assessing the impact of radiation risk feedback to clinicians on demand for CT scans [19]. The following message was added to all CT scan reports at a busy acute teaching hospital situated in a regional healthcare Trust in the UK:

*“Message from the executive medical director: “did you know that a chest, abdo and pelvis CT scan in a 20 year old female population is associated with approximately a 1 in 300 risk of subsequent cancer? The equivalent risk is much lower in 90 year old men (less than 1 in 3000). Is there an equally effective alternative investigation that does not involve ionising radiation? If so, have you discussed all of the alternatives with your patient?”* http://www.xrayrisk.com/index.php”

Before the intervention doctors at the hospital were invited to participate in an on-line survey of radiology knowledge. A targeted reminder email was sent to non-responders 8 weeks later. The survey asked doctors to indicate their grade, medical specialty and whether they had received formal radiation safety training. Multiple choice questions measured knowledge of the imaging modalities that involve ionising radiation and of the level of lifetime cancer risk associated with a chest, abdomen and pelvis CT scan for a 20 year old female. The exact risk from exposure to ionising radiation is unknown and contentious, and dependent on many variables. The estimated risk based on doses recorded on scanners at the hospital in 2015 was between 1 in 300 and 1 in 3000, so either of these two estimates were considered the best response. Two other response options represented an underestimation of risk (1 in 30,000 or negligible) and one represented an overestimation of risk (1 in 30), and so either of these responses were regarded as unambiguously wrong. Approximately 1 year after the baseline survey it was repeated with an additional question assessing whether doctors had noticed the intervention. The surveys were anonymous but as an incentive respondents could provide an email address to enter a prize draw to win an iPad.

Self-reported participant characteristics and awareness of ionising radiation risk before and after the intervention were compared using χ^2^ tests or Fisher’s exact tests if the assumptions of χ^2^ were not met. Association between doctor grade and awareness was tested for each survey using χ^2^ tests. A sensitivity analysis was used to explore doctors’ training and knowledge of the risks of radiation exposure, assuming all non-respondents had received training and had perfect knowledge. As these data formed part of an evaluation of health service delivery, approval from an institutional ethics committee or UK Research Ethics Service was not deemed necessary according to national regulations [20].

## Results

The number of doctors completing the survey was 170 at baseline (31.1% of 546 invited) and 168 at follow-up (19.5% of 863 invited). Samples were similar on medical specialty and receipt of formal radiation safety training but participants were significantly more likely at follow-up than at baseline to be consultant grade (Table 1).

**Table 1:** Self-reported characteristics of respondents to before and after surveys

	Baseline respondents (*N* = 170) n (%)	Follow-up Respondents (*N* = 168) n (%)	*P* value
What grade of doctor are you?			
F1 to F2	27 (15.9)	1 (0.6)	< 0.001
ST1 to ST3	27 (15.9)	18 (10.7)	
Training grade ST3+	27 (15.9)	19 (11.3)	
Consultant	78 (45.9)	107 (63.7)	
Other	11 (6.5)	23 (13.7)	
Which of these best describes your medical speciality?			
Medicine	50 (29.4)	44 (26.2)	0.355
Surgery	27 (15.9)	28 (16.7)	
Trauma and Orthopedics	8 (4.7)	12 (7.1)	
Obstetrics and Gynaecology	4 (2.4)	6 (3.6)	
Oncology/Haematology	3 (1.8)	8 (4.8)	
Pediatrics	14 (8.2)	7 (4.2)	
Radiology	9 (5.3)	16 (9.5)	
Anaesthetics	19 (11.2)	12 (7.1)	
Emergency Medicine	17 (10.0)	19 (11.3)	
Other	19 (11.2)	16 (9.5)	
Have you received formal training on radiation safety with regard to diagnostic investigations?			
Yes	111 (65.3)	106 (63.1)	0.673
No	59 (34.7)	62 (36.9)	
If yes, where was this?			
Medical school	44 (38.9)	42 (39.3)	0.962
After medical school	69 (61.1)	65 (60.1)	

At baseline 65% of respondents indicated they had received formal training on radiation safety with regard to diagnostic investigations (Table 1). Radiation training had been received by 81% of consultants, and 52% of training and foundation grade doctors. In the sensitivity analysis that assumed that all those who did not respond had actually received formal training in the risks associated with radiation exposure, 11% of the study population would still have not received any training. Of those who had received training, 39% received training at medical school and 61% after medical school (Table 1).

Proportions of doctors at baseline that identified specific imaging modalities that involve ionising radiation was very high for CT scans and chest x-rays and lower for isotope bone scans and positron emission tomography (PET) scans. A very small proportion of respondents incorrectly stated magnetic resonance imaging (MRI) scans and ultrasound scans involved exposure to ionising radiation (Table 2).

**Table 2:** Awareness and knowledge measures of all respondents before and after intervention

	Baseline respondents (*N* = 170) n (%)	Follow-up respondents (*N* = 168) n (%)	*P* value
Which of these imaging modalities involves ionising radiation (tick all that do so)?			
Ultrasound scan (USS)	6 (3.5)	7 (4.2)	0.761
Magnetic Resonance Imaging (MRI)	13 (7.6)	13 (7.7)	0.975
Computerised tomography (CT) scan	166 (97.6)	163 (97.0)	0.722
Chest x-ray	165 (97.1)	161 (95.8)	0.543
PET scan	137 (80.6)	139 (82.7)	0.610
Isotope bone scan	142 (83.5)	147 (87.5)	0.300
What is the average impact of a Chest, Abdomen and Pelvis CT scan on the lifetime risk of cancer for a 20 year old female?			
Associated with approximately 1 in 30 lifetime risk of subsequent cancer	8 (4.7)	2 (1.2)	<0.001
Associated with approximately 1 in 300 lifetime risk of subsequent cancer^a^	38 (22.4)	64 (38.1)	
Associated with approximately 1 in 3000 lifetime risk of subsequent cancer^a^	78 (45.9)	74 (44.1)	
Associated with approximately 1 in 30,000 lifetime risk of subsequent cancer	35 (20.6)	27 (16.1)	
Negligible	11 (6.5)	1 (0.6)	
Have you noticed radiation harm information below the CT report at [hospital name]?	Not asked at baseline		n/a
Yes		139 (83.7)	
No		27 (16.3)	

^a^Best responses

At baseline the level of lifetime cancer risk associated with a chest, abdomen and pelvis CT scan for a 20 year old female was identified as approximately 1 in 300 by 22.4% of respondents and approximately 1 in 3000 by 45.9% (Table 2). Approximately 5 % overestimated the risk (1 in 30) and 27.1% underestimated the risk (1 in 30,000 or negligible). In the sensitivity analysis, assuming that all doctors who did not respond had perfect knowledge on the topic, this would still give 8% of doctors who unambiguously underestimated the risk in the diagnostic test scenario presented. No association was found between grade (consultant or training/foundation level) and estimation of associated lifetime cancer risk at baseline (*Χ*^2^(4) = 0.829, *p* = 0.935) or follow-up (*Χ*^2^(4) = 1.1810, *p* = 0.881).

Following exposure to the intervention for 1 year there was a statistically significant improvement in respondent estimates of the long-term health impacts of ionising radiation exposure (*p* < 0.001); an increase from 22.4 to 38.1% of respondents estimating the risk as approximately 1 in 300 for a 20 year old female (Table 2). Only 1.2% overestimated the risk (1 in 30) and 16.7% underestimated the risk (1 in 30,000 or negligible). There was an absolute increase of 15.7% in the proportion giving the risk estimate that was cited in the educational message (1 in 300) and decreases in the proportions selecting all other responses. Overall the proportion with either of the two optimal responses increased from 68.3 to 82.2%. There was no change in the proportion of doctors correctly identifying imaging modalities that do or do not involve ionising radiation (Table 2). The proportion of respondents indicating they had noticed the educational message was 83.7% (Table 2).

In the subgroup in each sample that reported having not received formal training in radiation safety there was a statistically significant improvement in estimates of the long-term health impacts of ionising radiation exposure (*p* = 0.047). There was an absolute increase of 22.9% of these non-trained respondents providing one of the two best responses (Table 3). In the subgroup that had received formal training there was a statistically significant improvement (*p* = 0.008) and an absolute increase in best responses of 9.2%, leaving the untrained and trained subgroups with similar proportions of best responses at follow-up (Table 3).

**Table 3:** Awareness and knowledge measures in untrained and trained respondent subgroups before and after intervention

	Baseline untrained respondents (*N* = 59) n (%)	Follow-up untrained respondents (*N* = 62) n (%)	*P* value	Baseline trained respondents (*N* = 111) n (%)	Follow-up trained respondents (*N* = 106) n (%)	*P* value
Which of these imaging modalities involves ionising radiation (tick all that do so)?						
Ultrasound scan (USS)	4 (6.8)	2 (3.2)	0.432	2 (1.8)	5 (4.7)	0.271
Magnetic Resonance Imaging (MRI)	9 (15.3)	7 (11.3)	0.520	4 (3.6)	6 (5.7)	0.531
Computerised tomography (CT) scan	58 (98.3)	60 (96.8)	1.000	108 (97.3)	103 (97.2)	1.000
Chest x-ray	57 (96.6)	57 (91.9)	0.440	108 (97.3)	104 (98.1)	1.000
PET scan	42 (71.2)	52 (83.9)	0.094	95 (85.6)	87 (82.1)	0.482
Isotope bone scan	42 (71.2)	51 (82.3)	0.149	100 (90.1)	96 (90.6)	0.906
What is the average impact of a Chest, Abdomen and Pelvis CT scan on the lifetime risk of cancer for a 20 year old female?						
Associated with approximately 1 in 30 lifetime risk of subsequent cancer	3 (5.1)	1 (1.6)	0.047	5 (4.5)	1 (0.9)	0.008
Associated with approximately 1 in 300 lifetime risk of subsequent cancer^a^	12 (20.3)	21 (33.9)		26 (23.4)	43 (40.6)	
Associated with approximately 1 in 3000 lifetime risk of subsequent cancer^a^	24 (40.7)	31 (50.0)		54 (48.6)	43 (40.6)	
Associated with approximately 1 in 30,000 lifetime risk of subsequent cancer	14 (23.7)	8 (12.9)		21 (18.9)	19 (17.9)	
Negligible	6 (10.2)	1 (1.6)		5 (4.5)	0 (0.0)	

^a^Best responses

## Discussion

These data provide evidence that firstly, knowledge among doctors of the lifetime cancer risk associated with CT scans was low before the intervention. Secondly, a simple intervention over a 1 year period appears to have been effective at improving awareness of lifetime risks of exposure to CT scans, although findings are interpreted with caution due to potential confounders.

At baseline, 32% of respondents had limited knowledge of the health consequences of receiving a CT scan, and over a quarter of respondents underestimated the cancer risk of exposure to CT scans. A previous study from England found that 44% of respondents underestimated this risk, 50% identified the correct risk level and 6% overestimated the risk [11]. In an Australian study 78% underestimated and 5% overestimated the radiation dose from a chest CT scan and 10% thought there was no associated cancer risk [12]. In the USA 17% of emergency department providers (physicians, physician assistants and nurse practitioners) underestimated the risk of receiving a CT scan and 23% selected ‘don’t know’ [13], after an earlier study reported that 91% believed there was no increased risk [14]. Our study adds to a growing body of international evidence reporting a tendency for hospital doctors to underestimate the future health risks of CT scans. Thirty five per cent of respondents indicated they had not received formal training on radiation safety in diagnostic investigations. This is a concern and highlights the need to develop and evaluate new approaches to improving doctors’ knowledge that could prevent avoidable harm to patients.

Correct identification of imaging modalities that do or do not involve ionising radiation was higher in our study than in previous research. For example, approximately one third of respondents in a study from Hong Kong stated that PET scans and radio isotope scans do not involve radiation and a similar proportion stated that MRI scans do involve radiation [15]. In our data the proportions at baseline were 19, 17 and 8%, respectively. However, the figures from the previous study refer only to non-radiologists whereas 5% of our baseline sample and 10% of our follow-up sample were radiologists. The English study reported that 15% of respondents thought MRI utilises radiation [11], compared to only 8% in our study.

Awareness of the degree of risk associated with CT scans was significantly greater after exposure to a simple non-directional educational message for 1 year. Multifaceted programmes in the USA have been demonstrated to reduce use of CT scans in hospitals [21, 22]. However, the intervention often involves resource-intensive ongoing efforts to educate and change practice. Our study demonstrates the potential of a relatively economical intervention, using a light touch approach without reducing autonomy, to improve awareness in doctors.

The results emphasise an urgent need for strategies to improve awareness in this area and reduce use of avoidable CT scans. From a legal perspective this is important. The Ionising Radiation (Medical Exposure) Regulations 2017 (IRMER) state that the referrer has a responsibility to provide the practitioner sufficient information to justify the investigation [9]. In addition to this, guidance from the General Medical Council and the Society of Radiographers advise that the referrer should be able to discuss the risks and benefits of any investigation to the patient [7, 8]. Clearly this duty cannot be fulfilled if the referrer is unaware of the radiation dose of an investigation, or even whether the investigation involves ionising radiation at all.

A complicating factor may be the uncertainty over the risk posed by radiation exposure from medical imaging. The “linear no-threshold” (LNT) model is the most commonly used approach when estimating the risk of radiation doses less than 100 mSv. However, this is controversial with some observers suggesting the risks are overstated, particularly at lower doses of radiation exposure [23]. This model also applies to populations rather than individuals and there are numerous other factors that will influence both the dose and the effect of the exposure (for example the precise CT protocol and the age, sex and weight of the patient) which make accurately assessing an individual’s risk more challenging. Using these models and doses recorded on CT scanners at the intervention hospital in 2015, the correct radiation risk for a hypothetical patient of the lifetime risk of cancer that may be attributed to radiation exposure is between one in 300 and one in 3000. Even when allowing for this uncertainty, however, almost one third of our respondents provided alternative estimates outside of this range. Although the individual risk estimates are small relative to the background lifetime risk of cancer, the growth in use of CT scans generates public health concerns as individual risks are applied to an increasingly exposed population [5].

The study sample included doctors from a range of specialties, which did not differ significantly between the cross-sectional before and after samples. Some limitations of the survey should be taken into account. The follow-up sample included a significantly greater proportion of consultant grade doctors, however there was no association between doctor grade and knowledge of risk. Gadolinium contrast can be used in MRI scans and although non-radioactive, in certain patient groups should be used with caution, which may have influenced the 8% of our respondents that thought MRI scans involve ionising radiation exposure. The response rate was relatively low and those with greater knowledge about radiology tests may have been more likely to take part as they were aware of the topic of the survey. However, at least 11% of the study baseline sample had not received any training, demonstrating that this is a concern for the safe provision of clinical care.

The survey question about lifetime risk of cancer corresponded to the specific scenario presented in the intervention message. There was no change in awareness of imaging modalties that do and do not involve ionising radiation, so it is unclear what impact the intervention may have had on awareness of risk associated with diagnostic imaging beyond the given scenario. The longer term effects of the intervention are also unknown. Future studies should undertake longer-term follow-ups and employ strategies to improve response levels. Regular mandatory training on the side-effects of exposure to ionising radiation and the importance of adopting non-ionising alternative imaging modalities may be required to enable a sustained improvement in the education of the workforce, and hence promote the use of ionising radiation judiciously and optimally.

## Conclusions

The findings of this intervention study demonstrate that awareness of cancer risk associated with diagnostic radiation is inadequate among hospital doctors and suggest awareness can be improved by exposure to a simple non-directional message. The wider and longer term impact of this single approach is unknown, and we acknowledge that our message may have scope for optimisation and refinement. These data highlight an area where there is a need for strategies to address clinicians’ awareness of the long-term health impacts of exposure to ionising radiation. It is likely that a range of interventions may be beneficial, ensuring that education from medical school onwards is complemented by regular mandatory training for clinicians, supplemented by simple awareness enhancing messages such as we have used in this study. The ultimate aim is develop an evidence base that helps to ensure that the powerful diagnostic properties of ionising radiation are used optimally.

## Data Availability

The datasets that support the findings of this study are available from the corresponding author upon reasonable request.

## Abbreviations

CT: Computed tomography
IRMER: Ionising Radiation Medical Exposure Regulations
LNT: Linear no-threshold
MRI: Magnetic resonance imaging
PET: Positron emission tomography
USS: Ultrasound scan
